# Correlation of the 4977 bp mitochondrial DNA deletion with human sperm dysfunction

**DOI:** 10.1186/1756-0500-2-18

**Published:** 2009-02-04

**Authors:** Fotini Ieremiadou, George C Rodakis

**Affiliations:** 1 Department of Biochemistry and Molecular Biology, National and Kapodistrian University of Athens, Panepistimioupolis, 157 01 Athens, Greece

## Abstract

**Background:**

Several studies have examined the association between mitochondrial DNA (mtDNA) deletions, in particular the "common" 4977-bp deletion, and human sperm dysfunction, but have produced contradictory results.

**Findings:**

Here we show that PCR slippage and primer miss-match to nuclear DNA may lead to overestimates in the frequency of deletions. Our investigation resolves this issue and gives strong negative correlation between the proportion of the "common" deletion and sperm motility. Furthermore, for the first time, we present data which reinforce the hypothesis for a negative correlation between the mtDNA "common" deletion and fertilization efficiency of spermatozoa.

**Conclusion:**

The present analysis resolves several literature inconsistencies and opens the way for diagnostic use of the "common" deletion as a molecular indicator of sperm fertility potential.

## Background

In the past decade more than 200 point mutations have been identified in human mtDNA and 45 of them proved to be associated with disease [1]. Also, more than 100 deletions are known to be involved in diseases [2]. In particular, a 4977-bp deletion has been associated with a number of pathological phenotypes [3-7]. This deletion, also found to accumulate during aging [8], has been characterized as the "common" deletion and used as an mtDNA damage indicator [9]. The 4977-bp deletion occurs between two 13-bp direct repeats, located at nucleotides 8470–8482 and 13447–13459 respectively, and results in mtDNA molecules (ΔmtDNA4977) that lack all or part of a 12 gene cluster. Due to the loss of several vital OXPHOS genes, mtDNA deletions mainly affect high-energy demanding post-mitotic cells, such as brain, liver and muscles. Since spermatozoon movement also requires a great amount of energy, defects in mitochondrial respiratory function is assumed to cause a decline in motility and, consequently, decrease of fertility.

Several studies have examined the correlation of mtDNA deletions and sperm dysfunction, but have produced contradictory results. Kao et al. [10] observed a negative correlation between presence and proportion of the 4977-bp deletion and sperm motility. These authors also reported a significantly higher incidence of the deletion in patients with asthenospermia, oligozoospermia and primary infertility when compared to normal individuals. In contrast, later reports have not found a clear association between the "common" deletion and infertility categorisation of donors [11,12]. There is also no clear correlation between multiple deletions and male infertility. Some studies have found a higher incidence of multiple mtDNA deletions in sub-fertile or infertile men [12,13], while other studies found no such association [14]. These deletions were not fully characterized, since neither the precise breakpoints nor the sequence of the amplified products were determined.

Here we show that the inconsistencies in the literature are due to technical inaccuracies of used methods, including primer miss-annealing and PCR slippage. We have applied an improved PCR assay in 31 samples with normal and 83 samples with abnormal sperm parameters and obtained a clear negative correlation between presence of the "common" deletion and sperm motility.

## Methods

### Preparation of spermatozoa and extraction of total DNA

Semen samples were kindly provided by "Eugonia-Iatriki Erevna-IVF unit, Greece" and "Embioiatriki-IVF unit, Greece" and classified according to World Health Organization (WHO) criteria [15]. After the semen sample was liquefied at room temperature, the spermatozoa were fractionated by Percoll™ (Sigma-Aldrich) 45% and 90% gradient [16]. Spermatozoa were collected from each layer and washed three times with five volumes of PBS (phosphate buffer saline; 137 mM NaCl, 2.7 mM KCl, 10 mM Na_2_HPO_4_, 1.8 mM KH_2_PO_4_, pH 7.4) to remove percoll. In order to avoid contamination from other cells (such as lymphocytes and epithelial cells), the sperm sample was incubated, prior to DNA extraction, with 50 mM Tris-HCl buffer (pH 6.8) at 8°C for 20 min [17], a treatment that does not affect spermatozoa. Spermatozoa were collected by centrifugation at 1000-g for 5 min. The sperm pellet was immediately subjected to DNA extraction as described by Douris et al. [18].

### Qualitative and quantitative PCR assays, PCR conditions, cloning and sequencing of PCR products

Detection of mtDNA deletion was carried out by PCR amplification using primers that flank the deleted region [see Additional file 1]. With these primers, conventional PCR gives a product only from molecules bearing the deletion, because the fragment from the normal mtDNA is too large to be amplified. Normal mtDNA molecules were detected using a forward primer located within the deletion (4977Fi) and an external reverse primer. This method produces a single PCR product when the sample contains only normal mtDNA and two products if it contains normal and deleted mtDNA molecules. The relative amounts of deleted and normal mtDNA were calculated using PCR-based serial dilution method [19] in mixtures of known proportions of deleted and normal mtDNA [see Additional file]. PCR reactions were carried out in a 25-μl reaction mixture containing 200 μM of each dNTP, 0.5 μM of each primer, 1 unit of *Taq *DNA polymerase (Promega), 1.5–2 mM MgCl_2_, and 1×reaction buffer (provided by the company). Cycling conditions were: initial denaturation at 94°C for 2 min, followed by 25–30 cycles of denaturation at 94°C for 30 sec, annealing at 55°C to 63°C (depending on the primer pair) for 45 sec and extension at 72°C for 30–60 sec. Long PCR reactions were performed with primers Fvelo and Rvelo, or D6 and R10 [see Additional file] in a number of samples, the obtained products were separated in agarose gels and the appropriate bands were excised and purified using the PureLink Quick Gel Extraction Kit (Invitrogen), and either cloned or directly sequenced using a commercial outlet. Long PCR products produced from four different semen samples with primers D6 and R10 were subjected to southern blot analysis [see Additional file 1].

### Statistical analysis

Results were analyzed by the pair-wise Chi-square test of independence or, when the expected numbers are small (less than five), by Fisher's exact test of independence.

## Results and discussion

**Figure 1:**
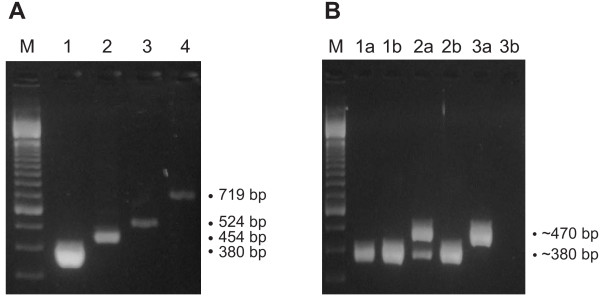
**Agarose gel electrophoresis of PCR products**. (A) Agarose gel electrophoresis of PCR products obtained by using four different primer sets in one of the examined semen samples. Lane M: 100-bp marker (Invitrogen, USA); lane 1: 380-bp PCR product (primers 4977F and 4977Rc); lane 2: 454-bp PCR product (primers 4977F and 4977R); lane 3: 524-bp PCR product (primers 4977F and 4977RK2); lane 4: 719-bp PCR product (primers 4977F and 4977RK3). Equal amount of each PCR reaction has been loaded to lanes 1 to 4. (B) Agarose gel electrophoresis of PCR products amplified from three different samples (1–3) with primers 4977F/4977Rc (lanes 1a, 2a, 3a) or 4977Fx/4977Rcx (lanes 1b, 2b, 3b). Lane M: 100-bp marker (Invitrogen, USA). Amplification with primers 4977F/4977Rc gave two products: a 380 bp band corresponding to ΔmtDNA4977 molecules (lanes 1a and 2a) and a ~470 bp band proved to be a by-product (lanes 2a and 2b). Specially designed primers 4977Fx/4977Rcx failed to produce the ~470 bp by-product (lanes 2b and 3b).

We tested 53 samples with normal and 96 samples with abnormal sperm parameters using the primer-shift PCR assay with previously published primer sets. We obtained a negative correlation between occurrence of the deletion and the size of the deletion product (r = -0.978, *P *= 0.021, Table 1). A similar observation was reported previously [20] suggesting that the larger fragments may lead to underestimation of the deletion occurrence, because they are less likely to be detected than the smaller fragments (Figure 1A). We conclude that the literature differences could be due to the different primer sets used.

**Table 1:** Occurrence of "common" deletion in human spermatozoa using different primer sets

			Occurrence of deletion	
			
#	Primer set	Product size	Our data^a^	Literature data
1	4977F – 4977Rc	380 bp	58.39% (87/149)	53.12% (34/64)^b^
2	4977F – 4977R	454 bp	42.95% (64/149)	56.55% (15/27)^c^
3	4977F – 4977RK_2_	524 bp	34.89% (52/149)	37.5% (15/40)^d^
4	4977F – 4977RK_3_	719 bp	17.45% (26/149)	Not available

^a ^PCR in 45% percoll fraction

^b ^PCR in non percoll fractionated sperm [11].

^c ^nested PCR with reverse primer MT13 [see supplementary Table 1 of Additional file] in 45% percoll fraction [12].

^d ^PCR in 50% percoll fraction [10].

Chi-square test between our data and the data from the literature gives *P *= 0.477 for primer set #1, *P *= 0.226 for primer set #2, and *P *= 0.760 for primer set #3

This hypothesis led us to the use of primers 4977F and 4977Rc, which produce the smallest deletion-specific fragment. PCR analysis in 61 of 149 samples revealed a larger product, in addition to the product expected from the presence of the "common" deletion, an observation that suggests the existence of a new deletion (Figure 1B). Isolation, cloning and sequencing of this PCR product showed that it corresponds to a region of the human chromosome 11 [see Additional file]. Similar by-products could also be produced by long PCR, as revealed by southern blot and sequencing [see Additional file]. Given that such by-products, which may easily result from the use of small primers, could be mistaken as deletions, is imperative to confirm the presence of any newly deletion-like product by sequencing. This is particularly so when using long-PCR for detection of multiple deletions, because it is customary to assume that all amplified products correspond to mtDNA deletions. We eliminated the miss-annealing by increasing primer size (Figure 1B). The new primer set (4977Fx/4977Rcx) also improved the sensitivity of the detection, since high melting point primers allow the use of large template amounts, which in turn allows the detection of deletions represent in low proportions [21].

To examine the possibility that the "common" deletion could be formed artificially as a result of repeated sequences [22-25], we used a cloned ~7 kb mtDNA fragment as template that did not contain the deletion ("intact" reference template, see Additional file) in primer-shift PCR reactions. These reactions gave a deletion-specific product, whose length depended on the primer set used. This result shows that a deletion-like PCR product does not necessarily signify the presence of a deletion. We assume that the pseudo-deletion (*ψ*-deletion) resulted from PCR-slippage. PCR analysis in a serially diluted sample of the "intact" mtDNA showed that the normal product was obtained for rather high dilutions of the "intact" mtDNA compared to the *ψ*-deletion product [see Additional file]. The ratio of the higher dilution that allows the amplification of the *ψ*-deletion and of the higher dilution that allows the amplification of the normal product (I_d_/I_n_) was 0.0013 (see "reference table", Table 2 of Additional file). This value of I_d_/I_n _is the threshold below which the deletion-specific product would not indicate presence of the deletion in the mtDNA of a sample.

**Table 2:** Proportion of the "common" deletion in 45% and 90% percoll fraction of semen samples

				Proportion of deletion^b^			
				
Percoll fraction	Sperm classification^a^		Number of samples	10% – 1%	1% – 0.1%	<0.1%	0%^c^
			31	1 (3.22%)	8 (25.81%)	3(9.68%)	19 (61.29%)
	
	Defected	A	10	5 (50%)	1 (10%)	0 (0%)	4 (40%)
		
		A	11	2 (18.18%)	4 (36.36%)	0 (0%)	5 (45.45%)
		
		T	5	0 (0%)	1 (20%)	0 (0%)	4 (80%)
		
		AT	20	8 (40%)	3 (15%)	0 (0%)	9 (45%)
		
		OA	7	2 (28.57%)	2 (28.57%)	0 (0%)	3 (42.85%)
		
		OT	5	0 (0%)	1 (20%)	1 (20%)	3 (60%)
		
		OAT	25	12 (48%)	2 (8%)	2 (8%)	9 (36%)
		
		Total	83	29 (34.93%)	14 (16.86%)	3 (3.61%)	37 (44.58%)
	
	Total		114	30 (26.32%)	22 (19.3%)	6(5.26%)	56 (49.12%)

90%	Normal		28	0 (0%)	4 (14.29%)	3 (10.71%)	21 (75%)
	
	Defected	A	7	1 (14.28%)	0 (0%)	0 (0%)	6 (85.71%)
		
		T	7	0 (0%)	2 (28.57%)	0 (0%)	5 (71.42%)
		
		O	0	-	-	-	-
		
		AT	11	1 (9.09%)	1 (9.09%)	0 (0%)	9 (81.81%)
		
		OA	2	0 (0%)	1 (50%)	0 (0%)	1 (50%)
		
		OT	0	-	-	-	-
		
		OAT	12	3 (25%)	0 (0%)	0 (0%)	9 (75%)
		
		Total	39	5 (12.82%)	4 (10.25%)	0 (0%)	30 (76.92%)
	
	Total		67	5 (7.46%)	8 (11.94%)	3 (4.48%)	51 (76.12%)

^a ^A, Asthenozoospermic; T, Teratozoospermic; O, Oligozoospermic; AT: Asthenoteratozoospermic; OA: Oligoasthenozoospermic; OT: Oligoteratozoospermic; OAT: Oligoastenoteratozoospermic

^b ^Determined according to supplementary Table 2 [see Additional file].

^c ^No deletion-specific product was detected, or the detected deletion-specific product represents pseudo-deletion (I_d_/I_n _≤ 0.0013).

Samples were categorized in two groups based on their sperm parameters. "normal" group includes samples with normal parameters while "defected" group samples with at least one abnormal parameter (according to WHO criteria).

### Sperm classification, fertilization outcome and proportion of the 4977 bp deletion

We first determined the occurrence of the deletion in 31 semen samples with normal ("normal" group) and 83 samples with at least one abnormal sperm characteristic ("defective" group). In many samples a deletion-specific product was detected, but the I_d_/I_n _ratio was below the threshold of 0.0013 (in "normal" samples 36.84% and 30% of the deletion-positives for the 45% and 90% percoll fraction respectively, while in "defective" samples 4.16% and 0% for the 45% and 90% percoll fraction respectively). These samples are either false positives or their deletion proportion is lower than the resolution of the detection method. These findings point out that a significant number of samples, especially from "normal" donors, could be mistakenly considered as bearing the deletion.

Our results (Table 2) revealed a clear correlation between percoll fraction and occurrence of the "common" deletion, which was stronger in the "defective" group (Chi square test of independence, *P *= 0.003 and *P *= 0.0008, respectively). On the other hand, the frequency of occurrence of the 4977-bp deletion did not differ significantly between "normal" and "defective" groups (in both percoll fractions). Fisher's test did not reveal a significant difference between the "normal" group and any of the 7 "defective" subgroups (*P *> 0.05). These results indicate that the deletion is detected more often in spermatozoa with low motility, an observation that concurs with the findings of Kao et al. [10]. Also, no statistically significant correlation was revealed between the occurrence of the "common" deletion and sperm classification (in both percoll fractions). These findings are in broad agreement with the studies of Cummins et al. [11] and John et al. [12], but contradict the results of Kao et al. [10] who reported a higher incidence of the "common" deletion in men with oligoasthenozoospermia. This might be due to the extremely small number of examined oligoasthenozoospermic samples in the report of Kao et al. [10] (one man with oligozoospermia and one with asthenozoospermia). The same authors reported a negative correlation between presence of the "common" deletion and primary infertility. Primary or secondary infertility could be due to a number of causes that do not necessarily affect sperm characteristics [26]. Attempts to correlate mtDNA deletions with sperm characteristics must include a significant number of semen samples, strictly classified according to their sperm parameters.

**Figure 2:**
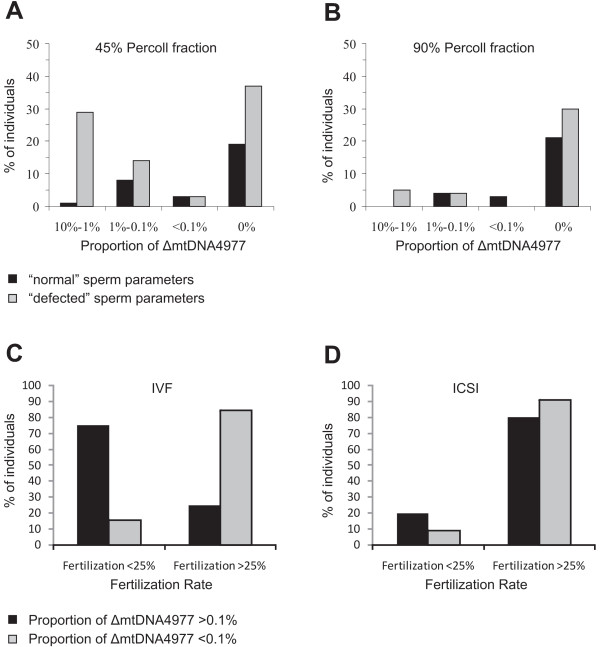
**Comparison of the "common" deletion and semen quality and fertilization ability**. (A-B) Comparison of the incidence of the "common" deletion in 45% and 90% percoll fraction from "normal" (A) and "defected" (B) semen samples. Samples were classified in four groups according to the calculated proportion of the deletion (see Additional file). (C-D) Comparison of the fertilization rate after IVF (C) and ICSI (D) from patients with different proportion of the "common" deletion. Samples were classified in two groups according to the calculated proportion of the deletion (above or below 0.1%).

Regarding the proportion of the "common" deletion, our results indicate that the majority of men with normal sperm parameters carried fewer ΔmtDNA4977 molecules compared to the "defective" group (*P *= 0.04) (Table 2 and Figure 2A, B). Fisher's exact test of independence also revealed that men with asthenozoospermia, asthenoteratozoospermia and oligoasthenoteratozoospermia carried more ΔmtDNA4977 molecules compared to "normal" individuals (*P *= 0.004).

According to WHO criteria, a "normal" semen sample could carry a number of spermatozoa with abnormal parameters, such us low motility or abnormal morphology. Fractionation of spermatozoa in percoll gradients separates the highly motile and morphologically normal spermatozoa [27,28], and under this explains the significantly higher occurrence of the deletion in the "low-motility" 45% fraction compared to the "high-motility" 90% fraction. Also, since the majority of spermatozoa with low motility, from "normal" or "defective" samples, congregate in the 45% fraction, we may explain the absence of a statistically significant difference in the occurrence of the deletion in this fraction between "normal" and "defective" samples. The same conclusion holds for the 90% fraction. Finally, our observation that "defective" samples bear a higher proportion of deleted molecules can be explained by the fact that "defective" samples carry more abnormal spermatozoa than the "normal" group.

Combining these findings with the observation that asthenozoospermic, asthenoteratozoospermic and oligoasthenoteratozoospermic men carried more ΔmtDNA4977 molecules, we conclude that there is a correlation between sperm motility, but there is no such correlation with morphology or sperm count, or with the proportion of the deletion. This could be explained under the assumption that mtDNA defects would primarily affect energy production and, thus, movement of spermatozoa.

From the 67 patients which underwent IVF or ICSI treatment, 14 had low fertility rates (<25%). Fisher's test revealed a significant correlation between the proportion of the "common" deletion in the 90% percoll fraction, and low fertilization rates after IVF (Figure 2C, D). Specifically, 75% of men that had a deletion proportion above 0.1% showed a low fertilization rate, in contrast to 15.64% with a deletion proportion below 0.1% (*P *= 0.0026). Nevertheless, no correlation was obtained between this parameter and fertilization rate in ICSI patients (*P *= 0.47). In IVF cases, sperm is selected by natural barriers, which means that spermatozoa with significant numbers of ΔmtDNA4977 molecules do not produce enough energy for their movement and thus oocyte fertilization is less likely to occur. ICSI, however, bypasses this natural selection process and allows fertilization by spermatozoa with low energy production. No statistically significant difference was detected between deletion proportion and pregnancy or delivery rates per transfer, after either IVF or ICSI. In other words our observations could imply that the load of common deletion in a semen sample reduces male fertility affecting mainly the fertilization efficiency of spermatozoa rather than affecting embryonic development. These results suggest, for the first time, a correlation between mtDNA integrity and fertilization efficiency of spermatozoa. *In vivo *studies in transmitochondrial mouse also underline the essential role of mitochondrial respiratory in mammalian spermatogenesis and demonstrate that mtDNA defects are responsible for male infertility [29].

The power of semen parameter analysis in predicting future fertility is questioned in the past few years [30]. Yet, new molecular tests, based on measurements of sperm DNA quality, aim at the successful treatment of male infertile patients. The mitochondrial genome of sperm has also been proposed as a marker of sperm health. Our investigation resolves several literature inconsistencies and points to a way for the construction of a molecular test for the estimation of sperm integrity and fertility potential, even in cases of idiopathic infertility.

## Competing interests

The authors declare that they have no competing interests.

## Authors' contributions

FI carried out the experiments and drafted the manuscript. GCR supervised the work, participated in experimental design and helped in manuscript preparation. Both authors contributed to data interpretation and analysis, and have read and approved the manuscript.

## Supplementary Material

Additional file 1 **Supplementary data.** Supportive information for the long-PCR experiments, Southern analysis and the quantitative PCR assay. Click here for file
