# Supplementary material for: Correlation of the 4977 bp mitochondrial DNA deletion with human sperm dysfunction
Source: BMC Res Notes. 2009 Feb 4;2:18. doi: 10.1186/1756-0500-2-18 (PMC2642850; doi:10.1186/1756-0500-2-18)
Supplement: Additional file 1 — Supplementary data. Supportive information for the long-PCR experiments, Southern analysis and the quantitative PCR assay. [file 1756-0500-2-18-S1.pdf]

ADDITIONAL FILE

Supplementary data

Evaluation of long-PCR specificity

Long-PCR analysis using primers D6 and R10 (John *et al.,* 2001; O' Connell *et al.,* 2002) resulted in the amplification of multiple PCR products which could indicate the presence of multiple mtDNA deletions within the examined region. In order to examine whether the non-expected bands (PCR-products) represent deletions in mtDNA, we applied the following assay:

1. Long PCR reactions were performed with primers Fvelo and Rvelo, or D6 and R10 (Suppl. Table 1 and Suppl. Fig. 1) in a 50 μl volume containing 200 μM of each dNTP, 0.3 μM of each primer, 1 unit of Elongase (Invitrogen), and 1 × reaction buffer B (provided by the company). Cycling conditions were: initial denaturation at 94°C for 2 min, followed by 30 cycles of denaturation at 94°C for 30 sec, annealing at 54°C or 65°C for 45 sec and extension at 72°C for 7–10 min (depending on primer pair).

2. Five μl of each of the long PCR products in three "defected" and one "normal" sperm sample was subjected to southern blot analysis. Following agarose gel electrophoresis, DNA was transferred onto a positively charged nylon membrane (Gene Screen Plus, New England Nuclear, USA) and hybridized with radioactive probes according to manufacturer's instructions. Radioactive mtDNA probes were obtained by nick-translation of two different gel purified PCR products. The first, ~8.7 kb, product was amplified by long PCR with primers D6 and R10, while the second, ~380 bp, product was amplified by standard PCR with primers Fsout and Rsout (Suppl. Table 1). The fragment, which amplified with the latter set of primers, was selected because it has no significant similarity to nuclear DNA sequences. Southern blot analysis showed that several smaller bands do not hybridized with either probe. BLAST search of the sequence of a cloned product (~1 kb) showed , that this product corresponded to nuclear DNA fragment (bases 62753 to 63756 of GenBank entry AC_020766.10; chromosome 16; algorithm, blastn; Identity = 99%; Expect = 0.0) (data not shown).

Quantification of the "common" deletion

Construction of "reference templates": Three different mitochondrial DNA fragments were cloned and used as "reference templates" (designated as "nor", "del" and "intact") in PCR reactions. The first fragment ("nor", 331 bp, corresponds to nucleotides 13178–13508 of the human mtDNA) was used to detect normal mtDNA molecules and was obtained by amplification using primers 4977Fi and 4977Rcx (Suppl. Table 1). Following amplification, the PCR product was cloned into the pCR2.1 vector (Invitrogen, USA) according to the company's instructions. Subsequently, recombinant plasmid was digested with *Eco*RI and the excised insert was separated and purified once more by agarose gel electrophoresis. The

same procedure was followed for the production of the reference template “del” after amplification with primers 4977Fx and 4977Rcx. The resulting “del” template represents a 386 bp fragment from mtDNA molecules bearing the “common” deletion (nucleotides 8146–8469 bp and 13447–13508 bp, concatenated). To construct the “intact” reference template, the ~7 kb product obtained from a long-PCR reaction with primers Fvelo, Rvelo was purified by agarose gel electrophoresis and cloned into TOPO-XL vector (Invitrogen, USA), according to the manufacturer’s instructions. The insert was then separated and purified once more by agarose gel electrophoresis after double digestion of the recombinant plasmid with *Kpn*I and *Not*I endonucleases.

Semi-quantitative PCR assay: Our technique was developed by improving the serial dilution method (Soong & Arnheim, 1996). Normal-specific and deletion-specific molecules were detected using two different forward primers (4977Fi and 4977Fx respectively) with similar characteristics, paired with the same reverse primer (4977Rcx), in order to avoid differences in product intensity due to variation in primer kinetics (Soong & Arnheim, 1996; Mehmet, 2001). Amplification was carried out for 25 cycles. Test PCR reactions carried out over a range of cycles, using serial dilutions of equimolar amounts of reference templates (“del” and “nor”), proved that at this number of cycles pairs 4977Fi/4977Rcx and 4977Fx/4977Rcx produced bands of equal intensity.

To avoid inaccuracies of the determination by eye of the dilution at which a product disappears (a major problem in the serial dilution method) we determined the end-point using Gel Analyzer software (Biosure Ltd, Greece). The estimated intensity of each PCR product was divided by the maximum intensity in the given series of dilutions (i.e. the PCR product intensity of the minimum template dilution). This ratio allowed intensity values to be adjusted on a scale from 0 to 1 (relative intensity). Relative intensity values of PCR products (x axis) were plotted against log values of template dilutions (y axis) (Fig. 2). The dilution at which the PCR product is no longer amplified, i.e. when $x = 0$, was determined by extrapolation (points I_n and I_d in Suppl. Fig. 2).

The relative amount of deleted molecules compared to normal molecules in a semen sample was calculated according to a “reference table” (Suppl. Table 2). This table was constructed using serial dilutions of a control DNA sample, which was a mixture of the “intact” DNA and seven different amounts of the “del” DNA representing 0%, 0.1%, 0.99%, 1.96%, 9.09%, 16.6% and 50% of the total DNA. The ratio of the highest dilution that allowed amplification of the deletion and the wild type mtDNA was used to construct the reference table.

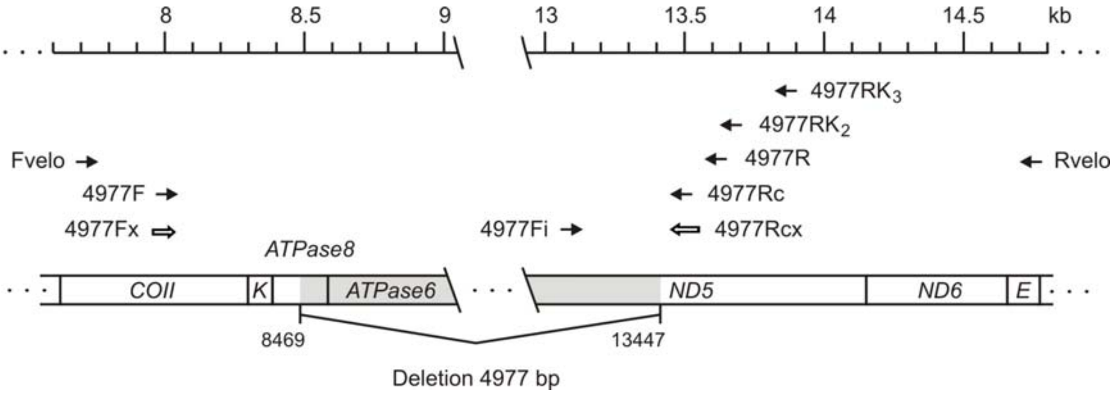

Supplementary Figure 1. Relative position and orientation of primers used in both conventional and long PCR. The primers 4977F, 4977Rc, 4977R, 4977RK2 and 4977RK3 were used for the detection of the 4977-bp deletion (shadowed area). Primers Fvelo and Rvelo were used to amplify, through long PCR, a ~7 kb region that included the area affected by 4977-bp deletion.

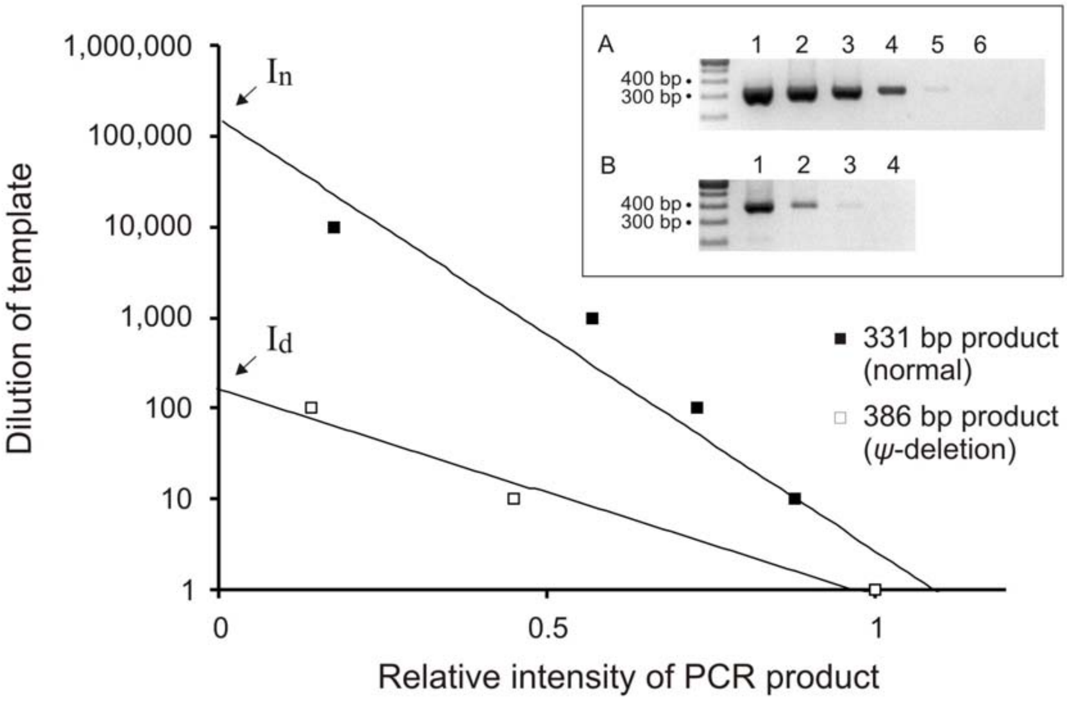

Supplementary Figure 2. Calculation of the proportion of ΔmtDNA4977 to normal mtDNA molecules using the serial dilution method. The y axis represents in logarithmic scale the dilution of the template, and the x axis represents the relative intensity of PCR product as calculated by Gel Analyzer. The dilution that the PCR product is no longer amplified, i.e. when x = 0, was determined by extrapolation (point I_n for normal PCR product and I_d for pseudo-deletion PCR product). Plots were constructed after agarose gel electrophoresis (top-right inset) of the 331-bp normal-specific (A) and the 386-bp pseudo-deletion (ψ-deletion) (B) products produced by PCR amplification of a 10 fold serially diluted “intact” reference template (lanes 1–6). The 331-bp and 386-bp products were amplified by PCR-primer pairs 4977Fi/4977Rcx and 4977Fx/4977Rcx, respectively.

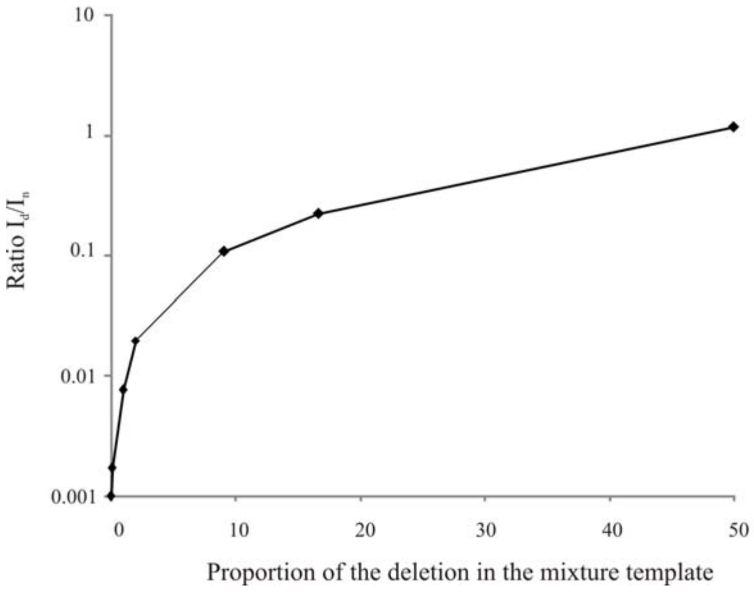

Supplementary Figure 3. Correlation between the calculated I_d/I_n ratio and the percentage of the "del" reference template in the "intact"-"del" mixture used for the construction of the reference table (Table 2) (r = 0.988).

```
PCR_product CCGGGGGTAT --ACTACGGT CAGT---TGG GAGATTACTT CCTTTACGTG TTTTTCTCTT GCCTCATCTT GAACTCCACC CCCATCAGAC ATACCGGTAT
Human_mtDNA .......... --........ ..A.GCTCT. A.ATC.GTGG AGCAA..CAC AG....ATGC C.A..G..C. AG.A.TA.TT ...C.A.A.A TCTTT.AA..
Human_nDNA  --..T.C... GT..C..... ....---... .......... .......... .......... .......... .......... .......... ..........

PCR_product GAGTGAAGTT AAGTCCAACA GACAGTGGCT CCAAGTCCTC AAGGACAACT AGGATTAATC ATTTTCCCTG TCCAATAATG AGTATTTGCA TGCATGCAAA
Human_mtDNA A---.GGCCC GTA.TT.C.C T.T..----- -..CCC.... T.CCC.CT.. ..AGCCC.CT G.AAAG.TAA CTT.GC.T.A .CCT...AAG .-----T...
Human_nDNA  .......... .......... .......... .......... .......... .......... .......... .......... .......... ..........

PCR_product GAGTGGCAGA GTTACAGCAT TTGTGGGGCA TATGGGTGTG GGCAGTGAAG GTGGGGTTTC CCTTAGATAA ACTCCTATAT GATGGGGCAT CAATATTTCC
Human_mtDNA ..T.AAG... ACC.ACA.C. C.T.A----- ---------- --.......A --------.G ..CC.AC... .-.A...CCG T....CC..C ..TA...---
Human_nDNA  .......... .......... .......... .......... .......... .......... .......... .......... .......... ..........

PCR_product AGGAAGCCGC ATTCTCCATA GAAACTATTG GTAAGGGGAG CTACTGGTCG TACAGCGGCA TGGTGCGGGT GCAGTGAGAG TGAAAGAGGG TAAGAGAACA
Human_mtDNA ----.C..C. ..A....T.. C.--....-- ---------- -.C..CA..A CC..A.TAA. AATATTAAAC A..----A.C .ACC.CCTAC CTCCCTC..C
Human_nDNA  .......... .......... .......... .......... .......... .......... ...A...... .......... .......... ..........

PCR_product GTAAAGAGAA AAATATGATA AGGGAGGGCC ATGGGGTTTT ACGATTTTAG TTA-CTTTCC TCACAGGTTT CT-AC 468
Human_mtDNA A.TGGC..CC T.GC..T.GC ...A.----- ---------- ---------- -..C...... .......... ..-.. 380
Human_nDNA  .......... .......... .......... .......... .......... ...-...... ......T.C. ..G.. 469
```

Supplementary Figure 4. Sequence comparison of the PCR product (produced with primers 4977F and 4977Rc, band ~500 bp, lanes 2a and 3a in Fig. 3b), and the corresponding sequences of human mitochondrial and nuclear DNA. The 380-bp “Human_mtDNA” sequence represents the expected PCR product produced from ΔmtDNA4977 molecules, using primers 4977F and 4977Rc. The “Human_nDNA” is the most similar to the “PCR_product” sequence, which returned by BLAST search (algorithm, blastn; Identity = 99%; Expect = 0.0) and corresponds to a fragment of human 11th chromosome (reverse complement of bases 66075 to 65619 of GenBank Acc. No. AC104009.7). The sequence corresponding to PCR primers is shadowed. Dots denote identical nucleotides and dashes denote gaps introduced for the alignment. Multiple alignment was performed by the program CLUSTALX v.1.83 (Thompson *et al.,* 1997) using the default parameters except for “Gap extension” which was set equal to 4. Minor corrections were made by eye inspection. Sequence comparison of the PCR product and the chromosomal region revealed 75% similarity for primer 4977F (with seven identical nucleotides at the 3′) and 90% for primer 4977Rc (with 12 identical nucleotides at the 3′).

Supplementary Table 1. Primers used for PCR amplification. “F” denotes forward and “R” reverse primer

Primer	F or R	Location[a]	Sequence	
4977F	F	8150-8166	5'-CCGGGGGTATACTACGGTCA-3'	Kao *et al.,* 1995; Cummins *et al.,* 1998
4977Rc	R	13506-13487	5'-GTAGAAACCTGTGAGGAAAG-3'	Cummins *et al.,* 1998
4977R	R	16580-16558	5'-GCGATGAGAGTAATAGATAGGGC-3'	Modified MT13 used by St. John *et al.,* 2001[b]
4977RK2	R	13650-13631	5'-GGGGAAGCGAGGTTGACCTG-3'	Kao *et al.,* 1995
4977RK3	R	13845-13826	5'-GTCTAGGGCTGTTAGAAGTC-3'	Kao *et al.,* 1995
4977Fi	F	13178-13198	5'-GCGCTATCACCACTCTGTTCG -3'	Modified MT13 used by St. John *et al.,* 2001
4977Fx	F	8146-8170	5'-ACGACCGGGGGTATACTACGGTCAATGC-3'	This study
4977Rcx	R	13508-13479	5'-GAGTAGAAACCTGTGAGGAAAGGTATTCCT-3'	This study
Fsout	F	16032-16058	5'-CTTTCATGGGGAAGCAGATTTGGGTAC-3'	This study
Rsout	R	16413-16390	5'-ACGGAGGATGGTGGTCAAGGGACC-3'	This study
Fvelo	F	7769-7795	5'-AAACCGTCTGAACTATCCTGCCCGCCA-3'	This study
Rvelo	R	14768-14739	5'-TAGTTTTGCGTATTGGGGTCATTGGTGTTC-3'	This study
D6	F	8286-8304	5'-TCTAGAGCCCACTGTAAAG-3'	St John *et al.,* 2001; O'Connell *et al.,* 2002; Reynier *et al.,* 1997
R10	R	421-403	5'- AGTGCATACCGCCAAAAGA -3'	St John *et al.,* 2001; O'Connell *et al.,* 2002; Reynier *et al.,* 1997

a In reference to the human complete mtDNA sequence, GenBank Acc. No. NC_001807.

b Primer was modified in order to have similar Tm with 4977F primer.

Supplementary Table 2. "Reference table" used for calculation of the proportion of ΔmtDNA4977. The Table was constructed by means of the calculated ratio I_d/ I_n when the percentage of the deletion is known in the original template mixture (ratio "del":"intact"). I_d/ I_n was calculated as the ratio of the highest dilution that allowed amplification of the deletion or pseudo-deletion (I_d) and of the normal (I_n) PCR product (see also supplementary Fig. 2)

Ratio "del":"intact" [a]	Proportion of the deletion in the template mixture	Average ratio I_d/ I_n [b]	Min ratio I_d/ I_n [b]	Max ratio I_d/ I_n [b]
0:1	0%	0.0010	0.0008	0.0013
1:1000	0.1%	0.0017	0.0014	0.0021
1:100	0.99%	0.0077	0.0063	0.0093
1:50	1.96%	0.0197	0.0161	0.0245
1:10	9.09%	0.1070	0.0863	0.1364
1:5	16.6%	0.2215	0.1557	0.3004
1:1	50%	1.1671	0.8648	1.5949

[a] Represents the molar proportion of the cloned fragments added in each PCR reaction; "del" is the 386 bp mtDNA fragment with the 4977-bp deletion, and "intact" is the ~7 kb fragment, which does not contain the deletion (see Materials and Methods).

[b] Each experiment repeated three times. For each triplet of I_d and I_n the average and the standard deviation (SD) were calculated. Max and Min I_d and I_n were calculated by adding or subtracting to/from the average two SD (Average ± 2×SD). Min (I_d/ I_n) ratio was calculated by dividing the Min I_d / Max I_n. Max (I_d/ I_n) ratio was calculated by dividing the Max I_d / Min I_n.
